# Poor long-term fellow-eye outcomes after vitrectomy with subretinal tissue plasminogen activator for AMD-related submacular hemorrhage

**DOI:** 10.1007/s00417-025-06944-0

**Published:** 2025-08-21

**Authors:** Shalev Fried, Ofri Vorobichik Berar, Daniel David, Mattan Arazi, Bar Klain, Gal Yaakov Cohen, Miri Fogel Levin, Eva Platner, Gabriel Katz, Avner Hostovsky

**Affiliations:** 1 Faculty of Medical and Health Sciences, Tel Aviv University, Tel Aviv, Israel; 2 The Goldschleger Eye Institute, Sheba Medical Center, Tel-HaShomer, Israel

**Keywords:** Submacular hemorrhage, Age-related macular degeneration, Tissue plasminogen activator, Pars plana vitrectomy, Fellow-eye

## Abstract

**Purpose:**

To evaluate the long-term prognosis of the fellow-eye in patients with age-related macular degeneration (AMD) who underwent pars plana vitrectomy (PPV) with submacular tissue plasminogen activator injection (SM-tPA) for significant submacular hemorrhage.

**Methods:**

Retrospective single-center cohort study including all consecutive patients who underwent PPV with SM-tPA for AMD-related submacular hemorrhage (SMH) between 2010 and 2022, with a minimum follow-up of 2 years. Data was collected from electronic medical records and included demographic characteristics, visual acuity, optical coherence tomography findings, adverse events, further ocular diagnoses in the fellow-eye.

**Results:**

Forty-seven patients (57% male; mean age 80 years) were included, with a mean follow-up of 82 months. At baseline, most patients had AMD in the fellow-eye (62% dry-AMD; 30% wet-AMD). During follow-up, AMD progressed in 64% of the patients who did not have wet-AMD in their fellow eye at the time of presentation. Seven patients (15%) experienced SMH in the fellow-eye within 1 month to 5 years after initial surgery.

The mean VA of the fellow-eye at presentation was 0.64 LogMAR, with a nonsignificant improvement in the first two years. Subsequently, a statistically significant VA deterioration was observed (final mean VA 0.8 LogMAR, *p*=0.009). By the final follow-up, 30% of patients had better VA in the operated eye, and 26% were legally blind in the fellow-eye.

**Conclusion:**

Patients treated with SM-tPA are at high risk for AMD progression and vision loss in the fellow-eye. Close monitoring of the fellow eye is warranted, and treatment decisions for the involved eye should not be based solely on good fellow-eye vision, given the long-term risk of deterioration.

## Key messages

*****What is known***:**

- Neovascular AMD often progresses bilaterally.
- Fellow-eye monitoring is standard in AMD care.

*****What is new***:**

- Over 80 months, 64% of fellow eyes progressed and 15% developed SMH.
- 26% of fellow eyes were legally blind at last follow-up.
- Long-term deterioration occurred despite initially good vision.

## Introduction

Submacular hemorrhage (SMH) is an uncommon sight-threatening complication of age-related macular degeneration (AMD). Less frequently, it can occur as a result of other ocular conditions such as retinal arterial macroaneurysms, pathological myopia, blunt trauma, and presumed ocular histoplasmosis syndrome [1, 2]. The natural history of SMH is usually of severe irreversible retinal changes leading to poor visual outcomes in most patients. Hemorrhage size and thickness, baseline visual acuity (VA), and delayed treatment were reported as poor prognostic factors [3–6]. Subretinal blood causes photoreceptor cell death by 3 mechanisms: iron toxicity, blockage of nutrient diffusion, and mechanical injury caused by fibrin clots [2].

There are no standardized treatment guidelines for SMH; Both surgical and non-surgical treatment options are employed aiming to displace the hemorrhage from the fovea, and the optimal strategy remains to be an enduring debate [7–9]. The main approaches include pars plana vitrectomy (PPV) as a standalone or with subretinal or intra-vitreal injection of tissue plasminogen activator (t-PA) and air-fluid exchange [8, 10–15], pneumatic displacement as a standalone or with intravitreal t-PA [8, 10, 16–18], and intravitreal anti-vascular endothelial growth factor (VEGF) antibodies injection [10, 19, 20]. Despite initial VA improvement following surgery, most patients continue to experience poor vision in the affected eye [12, 21–23], leading some ophthalmologists, due to the malignant nature of the disease, to advocate against surgical intervention. This is strengthened by the relatively high rate of surgical complications including cataract formation, retinal detachment, development of a macular hole, recurrence of SMH, and vitreous hemorrhage [8, 24].

A recent retrospective study reported that visual prognosis of eyes with best-corrected visual acuity (BCVA) below 20/200 was dismal with either vitrectomy or pneumatic displacement [25]. Thus, its authors suggested the following considerations in treatment choice: patient age, daily activities, wishes, presence or absence of vitreous hemorrhage, and the status of the fellow-eye. AMD is mostly a bilateral asymmetrical disease [26]. Good vision in the fellow-eye was suggested as a reason for delay in presentation and treatment in SMH cases [3]. Although the fellow- uninvolved- eye remains at high risk for developing advanced AMD [9], the baseline characteristics and prognosis of the fellow-eye are mostly not depicted in studies evaluating different treatment strategies. Here, we aim to describe the prognosis of the fellow-eye in patients treated with PPV and t-PA for AMD associated macular hemorrhage.

## Methods

This is a retrospective study evaluating electronic medical charts of all consecutive patients who underwent PPV with subretinal injection of t-PA for treatment of AMD-related SMH at the Chaim Sheba Medical Center, Israel, between 2010 and 2022. Inclusion criteria were adult patients with AMD-related SMH that were treated with the above-mentioned surgery and had a minimal follow-up period of 2 years. Patients who completed follow-up elsewhere and those with no light perception in the fellow-eye at presentation were excluded. At our center, SMH smaller than 4 optic disc diameters is not considered an indication for surgery, and thus, by institutional protocol, all included cases had SMH greater than this threshold. All patients underwent 23G or 25G vitrectomy with subretinal t-PA injection of 25 micrograms/0.1CC, followed by fluid air exchange. Subretinal t-PA is injected at our center using a 39G tip needle connected to a microdose 1 ml syringe (MedOne, Sarasota, Fl) and a viscous fluid injection set of the constellation machine.

Data collected included demographic characteristics, anticoagulation therapy, visual acuity, date of surgery, adverse events, further ocular diagnoses in the fellow-eye. BCVA was evaluated using the Snellen visual acuity (VA) chart and the data was converted to the logarithm of the minimum angle of resolution (logMAR) scale. When a chart was not visible by the patient from 1 m, VA was measured by counting fingers, hand movement, or by perception or no perception of light, and was converted to a logMAR values of 1.8, 2.3, 3.7, or 4.7, respectively. For the purpose of analyzing AMD progression in the fellow eye, we excluded patients who already had a diagnosis of wet AMD in the fellow eye at the time of SMH surgery in the first eye. AMD progression was defined relative to the AMD status documented at the time of surgery.

Categorical variables were described by frequency and percentage. Continuous variables were summarized by average and its standard deviation (SD). Comparisons of VA at different time points was performed using a paired Student’s *t*-test. All p-values were two-sided, and *p* < 0.05 was considered statistically significant. Data were analyzed using SPSS version 25.0 (SPSS Inc, Chicago, IL, USA).

## Results

Between 2010 and 2022, 58 patients underwent PPV + subretinal t-PA injection at our center for AMD-related submacular hemorrhage. Of those patients, 47 patients were included in the analysis and 11 patients were excluded: 2 patients died before completing a 2-year follow-up period, 8 patients continued their follow-up elsewhere, and 1 patient had no light perception in the fellow-eye at presentation.

Table 1 presents demographic and clinical Data of the included patients. The mean VA of the involved eye at presentation was 1.6 LogMAR (SD 0.55) and the mean size of the SMH at presentation was 13 optic disc diameter (SD 4.7 optic disc diameter). All SMH treated in this study were greater in size than 4 optic disc diameter. The mean follow-up time was 82 months (SD 47). Most of the patients had AMD diagnosis also on the fellow-eye upon examination at presentation (dry AMD, 62%; wet AMD, 30%). No patient developed macular hole during follow-up in the operated eye. Two patients had retinal tears identified and treated intraoperatively. Retinal detachment occurred in two patients in the operated eye: one 11 years after surgery following cataract extraction, and the other 7 years after surgery.

**Table 1:** Patient demographics and disease characteristics

Characteristic	*N* = 47	
Sex: male (%)	27 (57%)	
Age- mean (SD)	79.9 (7.8)	
Fellow eye (right) (%)	22 (47%)	
Phakic status of fellow eye (%)		
Phakic	13 (28%)	
Pseudophakic	34 (72%)	
Fellow-eye diagnoses at presentation^**┼**^ (%)		
Dry AMD	29 (62%)	
Wet AMD	14 (30%)	
Bullous keratopathy	1 (2%)	
Mean BCVA of fellow eye at presentation, logMAR (SD)	0.62 (0.61)	
Fellow eye BCVA below 20/200 at presentation (%)	8 (17%)	
Anti coagulation therapy	9 (19%)	

^**┼**^ Fellow eye clinical diagnosis at time of Submacular surgery at first eye

*AMD* age-related macular degeneration, *BCVA* best-corrected visual acuity

Twenty-one of the 33 patients (64%) whose fellow eyes did not have wet AMD at the time of SMH surgery, later developed wet AMD requiring anti-VEGF injections. The mean time to conversion was 32.1 months (SD 26.9) following surgery on their first eye. In addition, 1 (2%) patient developed central retinal vein occlusion. Notably, 7 patients (15%) developed SMH also in the fellow-eye, of which 3 patients (6%) underwent surgery, and the remaining 4 patients were treated with intravitreal anti-VEGF injections. Patients on anticoagulation therapy had a non-significantly higher proportion of SMH in the fellow-eye compared to those not on anticoagulation (3/9 [33.3%] vs. 4/38 [10.5%], Pearson Chi-Square *p* = 0.08). While this trend suggests a potential association, the small sample size limits definitive conclusions. Furthermore, the proportion of SMH treated with SM-tPA was significantly higher among patients on anticoagulation (2/9 [22.2%] vs. 1/38 [2.6%], Pearson Chi-Square *p* = 0.03)

**Fig. 1:**
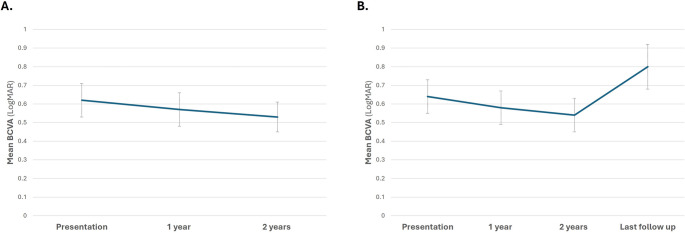
Changes in the mean visual acuity of the fellow-eye following submacular hemorrhage surgery. (**A**) The whole cohort during the first two years, and (**B**) all patients with follow-up longer than 2 years (45 patients). Error bars represent the standard error of the mean

The mean VA of the fellow-eye at presentation was 0.64 LogMAR (SD 0.62; 20/87). During the first two years following surgery there was a trend towards improvement in the VA of the fellow-eye (mean BCVA at 2 year 0.54 LogMAR [SD 0.59; 20/69], *t*-test *p* = 0.1). However, in an analysis among the patients who have had follow-up longer than 24 months (45 patients), a statistically significant decline in VA can be noticed after two years with a mean BCVA at last follow-up of 0.8 LogMAR [SD 0.8; 20/126], *t*-test *p* = 0.009; (Fig. 1).

**Fig. 2:**
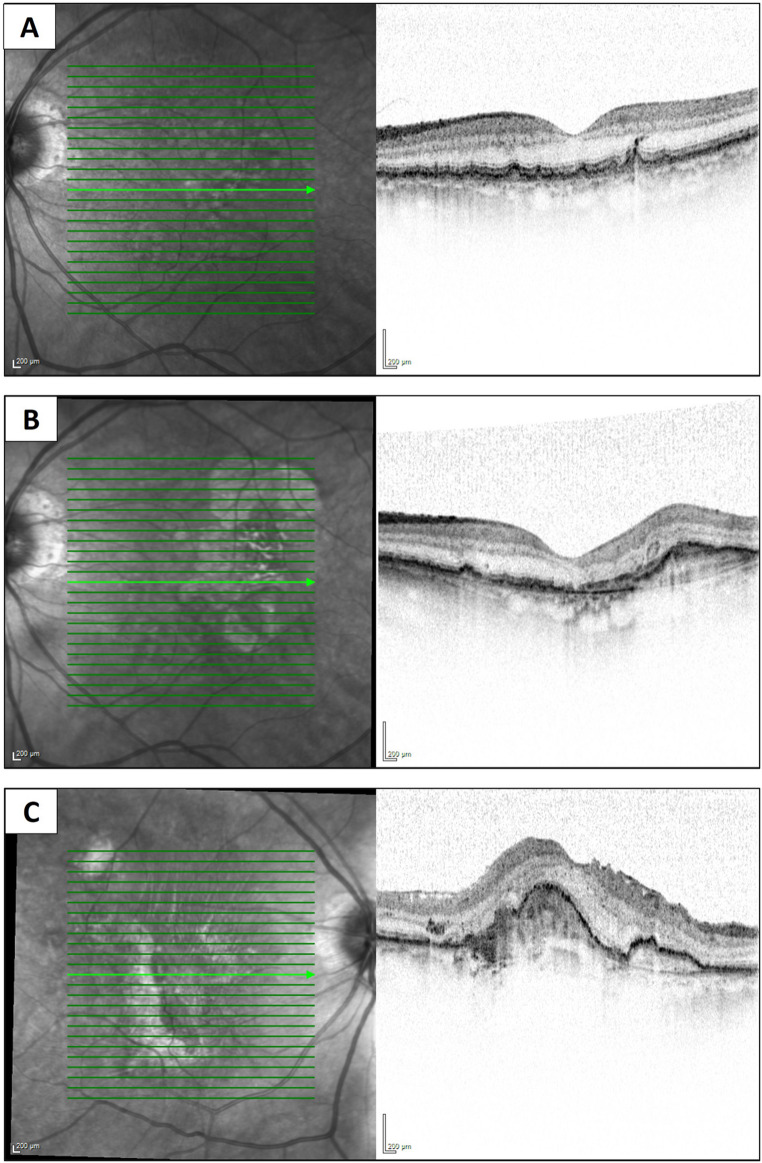
Near-infrared and SD-OCT images of an 85-year-old male patient with submacular hemorrhage included in the study cohort. (**A**) The fellow eye at the time of submacular hemorrhage diagnosis, showing a normal foveal contour with soft macular drusen. Visual acuity was 20/28. (**B**) OCT scan of the fellow eye at the last follow-up, three years post-surgery. During the first year after surgery, the patient developed choroidal neovascularization, requiring intravitreal bevacizumab injections. Visual acuity decreased to 20/60 at the last follow-up. (**C**) The involved eye with submacular hemorrhage at the last follow-up, with a visual acuity of 20/100

By the last follow-up, 7 (15%) patients experienced severe vision loss (defined as the loss of 0.3 logmar) and 12 (26%) patients were legally blind in the fellow-eye with VA lower than 20/200. Furthermore, at the last follow-up, 14 (30%) patients had a better VA in the operated eye compared to the fellow-eye (Fig. 2).

## Discussion

This study reports the outcomes of the fellow-eye in patients with AMD-related SMH who underwent PPV with subretinal tPA injection. To our knowledge, this is the first study that presents the long-term prognosis of the fellow-eye.

While fellow-eye monitoring is a cornerstone of AMD management, this study provides specific, long-term data on a unique subgroup: patients undergoing surgical treatment for SMH, a subgroup rarely evaluated in isolation. In our cohort, 64% of fellow eyes progressed to wet AMD, underscoring the ongoing risk in this population. For comparison, the SEVEN-UP [27] study, which followed 65 patients treated with intravitreal ranibizumab over seven years, reported a 31% progression rate to exudative AMD in the fellow-eye. Furthermore, a study describing the 96-week prognosis of the fellow-eye in subjects who were included in the post hoc analyses of the VIEW studies reported a 24% rate of AMD progression to exudative AMD [28]. Finally, Sadeghi et al. recently reported a 42% conversion rate over 60 months in a large cohort of patients with initially unilateral wet AMD [29]. Although our sample size and the lack of a control group limit direct comparisons, the relatively high fellow-eye progression and SMH rates observed in this cohort raise the possibility that patients presenting with AMD-related SMH may be at elevated risk for bilateral disease progression. Noteworthy is that 15% of the patients in our cohort developed SMH also in the fellow-eye. For comparison, data from the “Fight Retinal Blindness!” registry, which followed AMD patients receiving intravitreal anti-VEGF injections, suggest an estimated 10-year rate of SMH of 4.4% of patients [1]. Close follow-up of the fellow-eye is crucial for early identification of these complications. Additionally, we suggest that caution should be exercised with the “treat-and-extend” approach in the fellow-eye, given the potential for aggressive disease behavior in this subgroup.

Our findings indicate a progressive decline in VA in the fellow-eye over time. This deterioration was notable after an initial improvement during the postoperative period. It is likely that during this time, patients were in close follow-up by an ophthalmologist, underwent cataract surgery if needed, and received treatment for AMD. However, beyond 2 years, there was a gradual decrease in VA, highlighting the progressive nature of AMD and aligning with existing literature on the bilateral asymmetrical nature of AMD [26, 30]. By the last follow-up, 26% of patients were legally blind in the fellow-eye, compared to 17% at presentation. Additionally, 30% of patients had better VA in the operated eye compared to the fellow-eye at the last follow-up. This may reflect either the aggressive AMD features in the fellow-eye of those with SMH or the relative success of the surgical intervention in the treated eyes. The relatively late deterioration in visual acuity emphasizes the importance of long-term follow up studies in those cases. While our Data suggest that good baseline fellow-eye vision should not deter treatment in the affected eye, we acknowledge that our retrospective design does not allow conclusions regarding the superiority of surgical versus conservative management. This critical question is the focus of the ongoing TIGER trial — a phase 3 randomized controlled study comparing vitrectomy with subretinal tPA and gas versus anti-VEGF monotherapy [31]. Until those results are available, the choice of intervention should be individualised, considering each patient’s functional status, disease characteristics, and fellow-eye condition [32].

While our results suggest a possible association between anticoagulation therapy and SMH in the fellow-eye, these findings should be interpreted with caution given the limited sample size. We did not differentiate between anticoagulation subtypes due to the small numbers, and the observed differences did not reach robust statistical significance. Further studies with larger cohorts are needed to clarify the role of different anticoagulation in those patients.

This study has several limitations. First, information bias may affect the results due to the retrospective design of the study. Furthermore, although the number of included patients is relatively low, this reflects the rarity of SMH as a surgical indication. However, to our knowledge, this remains one of the largest long-term datasets specifically examining fellow-eye outcomes after vitrectomy with subretinal tPA. Finally, the exclusion of patients who completed follow-up elsewhere and those who died before the two-year follow-up period could have biased the results. Nevertheless, most of the patients who completed follow-up elsewhere are members of a specific healthcare provider, and we do not believe the reasons they continued follow-up elsewhere were specific characteristics they possess. Future studies with larger, multicenter cohorts are needed to confirm our result.

In conclusion, patients with AMD-related submacular hemorrhage who undergo PPV with subretinal tPA are at high risk of AMD progression and vision loss over time in the fellow-eye. The data presented here suggest that ophthalmologists should prompt close monitoring of the fellow-eye in the years following surgery.

## Abbreviations

AMD: Age-related macular degeneration
SMH: Submacular hemorrhage
PPV: Pars plana vitrectomy
t-PA: Tissue plasminogen activator
VA: Visual acuity
VEGF: Vascular endothelial growth factor
LogMAR: Logarithm of the minimum angle of resolution
